# Supplementary figures and images for: Profiling the Spatial Expression Pattern and ceRNA Network of lncRNA, miRNA, and mRNA Associated with the Development of Intermuscular Bones in Zebrafish
Source: Biology (Basel). 2022 Dec 31;12(1):75. doi: 10.3390/biology12010075 (PMC9855694; doi:10.3390/biology12010075)

# mRNA

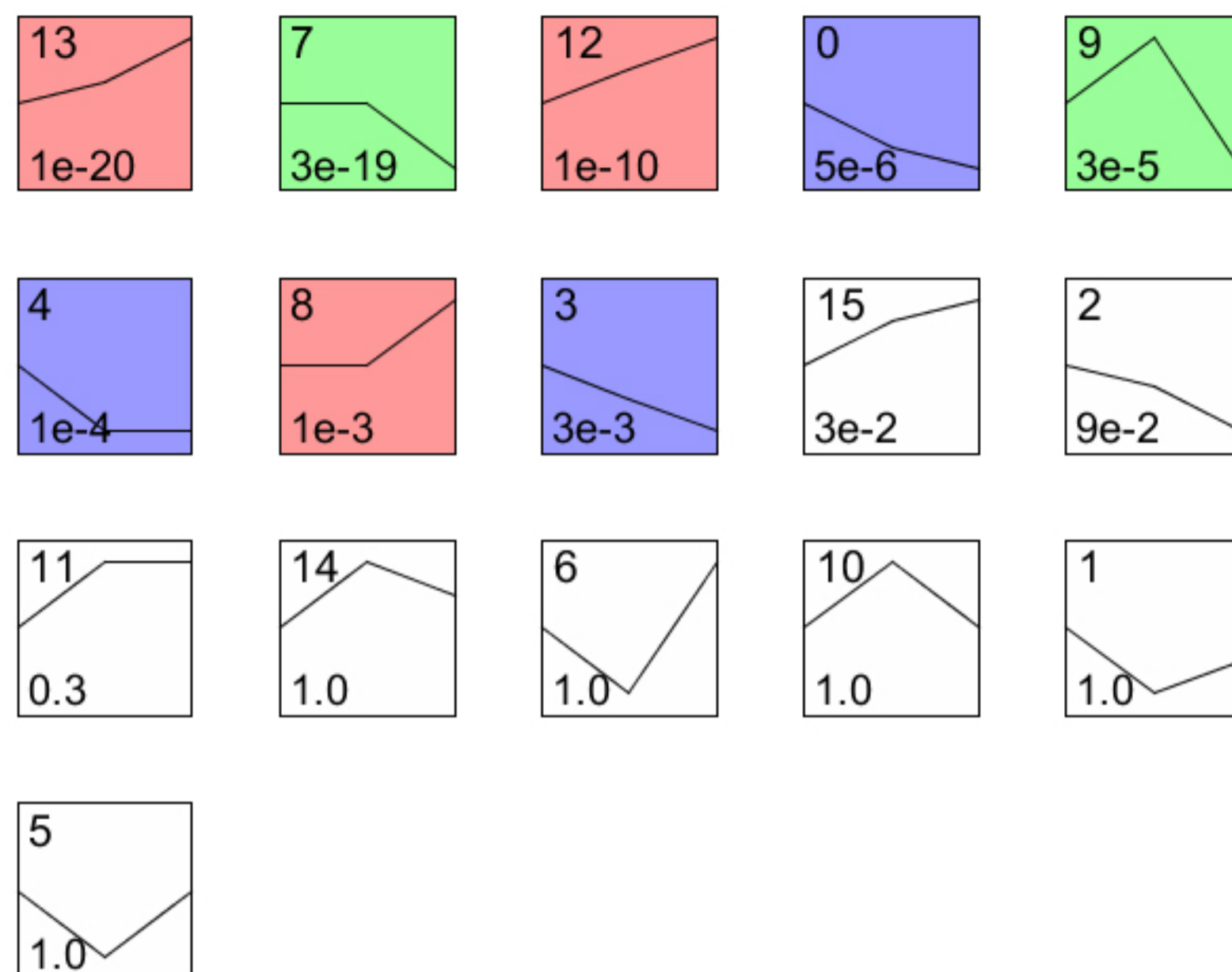

# miRNA

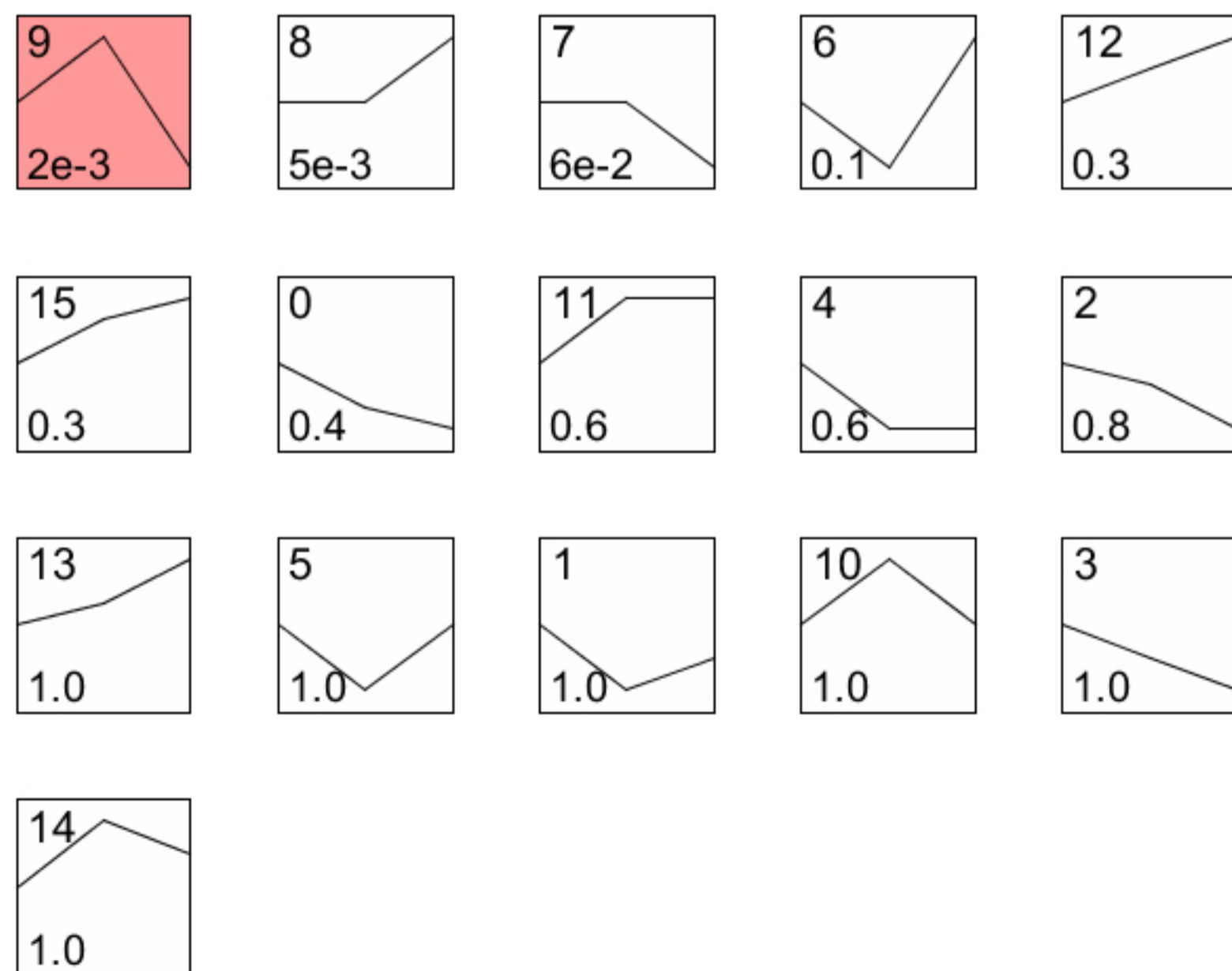

# lncRNA

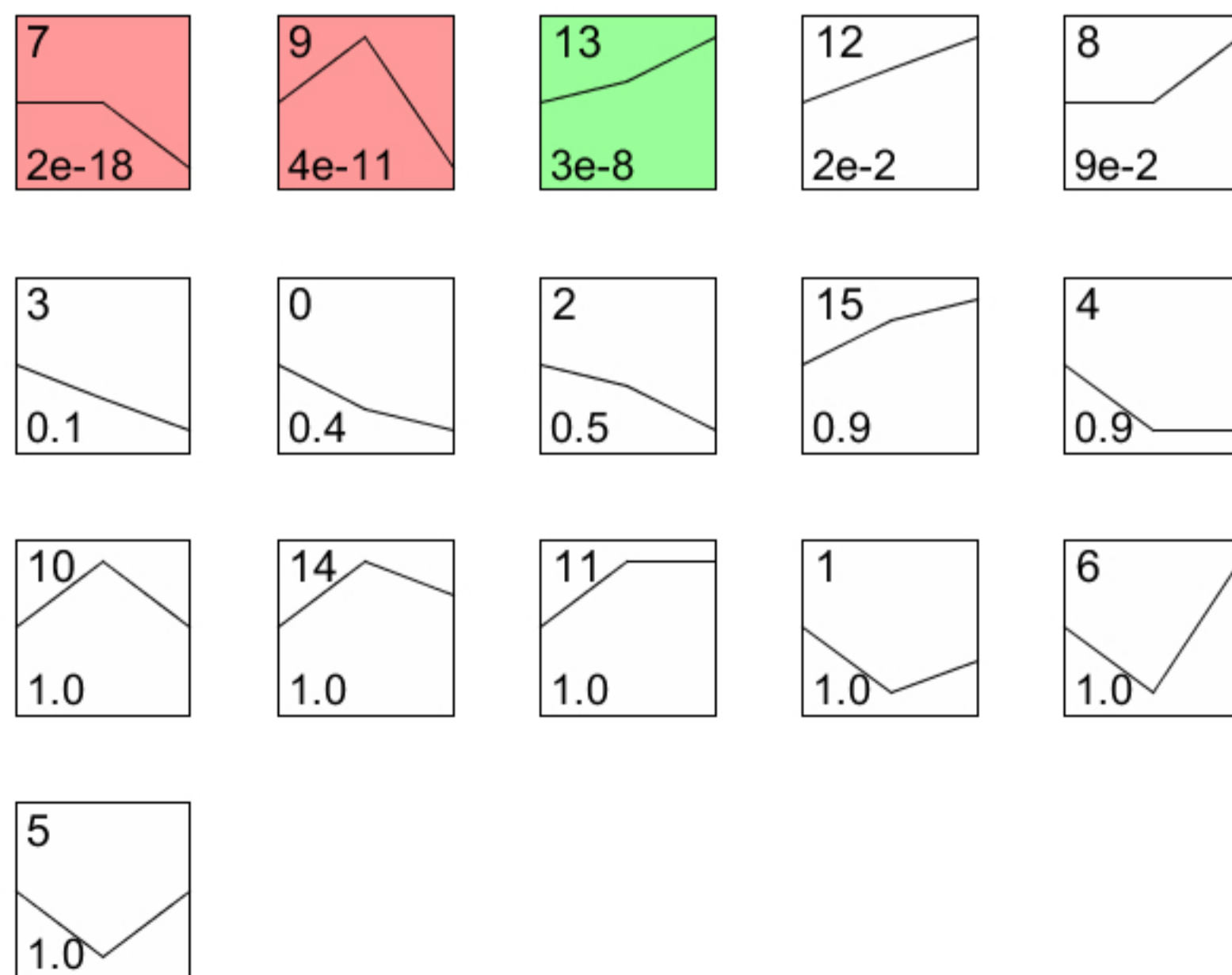

Supplement: Supplementary file 1 [file biology-12-00075-s001.zip › Figure S1.pdf]
